# Inactivation kinetics of 280 nm UV-LEDs against *Mycobacterium abscessus* in water

**DOI:** 10.1038/s41598-023-29338-w

**Published:** 2023-02-07

**Authors:** Jack Jia Xin Song, Kumiko Oguma, Satoshi Takizawa

**Affiliations:** Department of Urban Engineering, The University of Tokyo, Tokyo, 113-8654 Japan

**Keywords:** Civil engineering, Pathogens, Water microbiology

## Abstract

Nontuberculous mycobacteria (NTM) are opportunistic premise plumbing pathogens (OPPPs) that cause a burdensome waterborne respiratory disease. Due to their resistance to chemical disinfectants and regrowth in biofilms in drinking water distribution systems, treatment can be better performed using small ultraviolet disinfection units at the point-of-use (POU), such as at a tap or showerhead. Ultraviolet light-emitting diodes (UV-LEDs) are well suited for such applications, but fluence-response data are not available for one of the most important NTM, *Mycobacterium abscessus*. In this study, a bench-scale 280 nm UV-LED apparatus was used to irradiate *M. abscessus* in a water matrix. The fluence-response profile was sigmoidal, exhibiting both shoulder and tailing phenomena. Simple linear regression and the Geeraerd’s inactivation kinetics model yielded *k* values of 0.36 and 0.37 cm^2^/mJ, respectively, revealing that *M. abscessus* is more resistant to UV than *Pseudomonas aeruginosa* and *Legionella pneumophila*, which suggests that NTM are among the most UV-resistant OPPPs. Results of this study suggest that 280 nm UV-LED irradiation can be an effective and practical option to inactivate *M. abscessus* at the POU. Disinfection units that can deliver a fluence of 10 mJ/cm^2^ are expected to achieve nearly 2 log (99%) inactivation of *M. abscessus*.

## Introduction

Globally in 2020, 2 billion people still lack access to safely-managed drinking water services, and microbiologically contaminated drinking water is estimated to cause 485,000 diarrheal deaths each year^1^. Even in mature drinking water systems with performant disinfection processes, there has been an increase in outbreaks due to opportunistic premise plumbing pathogens (OPPPs) (also called biofilm-associated pathogens), mainly consisting of nontuberculous mycobacteria (NTM), *Legionella spp.*, and *Pseudomonas spp.*, which can dominate due to the selective pressure in drinking water treatment and distribution system conditions^2^. These pathogens are resistant to chemical disinfection, can associate with free-living amoeba, and have been shown to grow throughout water distribution systems and especially in premise plumbing. There, long residence times, low residual disinfectant levels, and insufficient hot water temperatures allow OPPPs to amplify and persist in biofilms. Clinically, OPPPs have been linked to outbreaks and provide a persistent exposure source, especially for vulnerable individuals. For these reasons, species of the three genera above have joined the United States Environmental Protection Agency’s (US EPA) Fifth Drinking Water Contaminant Candidate List (CCL 5)^3^, a “list of contaminants that are currently not subject to any proposed or promulgated national primary drinking water regulations, but are known or anticipated to occur in public water systems.”

To identify the most significant waterborne pathogens, Collier et al.^4^ reviewed 17 infectious waterborne diseases in the USA and found that while the OPPPs (NTM, *Pseudomonas*, *Legionella*) did not account for the most cases, they caused most of the hospitalizations and deaths. Among a total of 7,150,000 domestically acquired waterborne disease cases, those caused by OPPPs were estimated at 68,900 (NTM infection), 15,900 (*Pseudomonas* pneumonia), and 11,000 (Legionnaires’ disease). OPPPs caused notably high hospitalization rates of 74.8% (NTM infection), 97.2% (*Pseudomonas* pneumonia), and 98.1% (Legionnaires’ disease). NTM infection caused the most hospitalizations (51,400), followed by otitis externa (23,200), and *Pseudomonas* pneumonia (15,500). Among a total of 6,630 deaths, OPPPs again dominated, estimated to account for 3,800 (NTM infection), 730 (*Pseudomonas* pneumonia), and 995 deaths (Legionnaires’ disease). Overall, waterborne transmission causing respiratory disease accounted for 83% of all domestically acquired waterborne deaths. The total direct healthcare cost of waterborne disease in the US was estimated at $3.33 billion, with the three OPPP-related diseases (representing domestically-acquired waterborne respiratory diseases) accounting for $2.39 billion (71.8%). NTM infection was the costliest disease overall ($1.53 billion), followed by otitis externa ($564 million), and *Pseudomonas* pneumonia ($453 million). In contrast to historical studies prior to widespread water treatment and sanitation, Collier et al. results suggest that today’s waterborne disease burden comes primarily from environmental pathogens (e.g., OPPPs), as opposed to enteric pathogens that can be readily treated before distribution.

In light of their significant healthcare cost/burden and rising incidence rates, NTM were selected as the focus of this study. NTM are mycobacteria other than those that cause tuberculosis or leprosy. They are divided into slow-growing mycobacteria (SGM) and rapid-growing mycobacteria (RGM), generally represented clinically by *M. avium* complex (MAC; consisting of *M. avium*, *M. intracellulare*, and *M. chimaera*) and *M. abscessus* complex (MABS; consisting of *M. a. abscessus*, *M. a. massiliense*, *M. a. bolletii* subspecies), respectively. The relative prevalence of the two types is related to geography—RGM account for 10–20% of NTM isolates worldwide but as much as 50% in parts of East Asia^5^. Both are important drinking water contaminants—MAC has been part of the US EPA’s CCL since CCL 1 (1998), while *M. abscessus* was recently added to CCL 5. NTM infection most commonly manifests as chronic pulmonary disease^6^, and epidemiological studies from North America, Europe, Asia, and Australia have all found increasing NTM incidence rates over the past 2 decades^7^. Treatment is difficult and requires multi-drug therapy for long periods (sometimes years), and surgery may be necessary^8^. Natural and drinking waters form the main source of human infection with NTM^9^. NTM are opportunistic pathogens and can form hardy biofilms and persist in moist environments, such as municipal water distribution systems and household plumbing systems^10^. While it is possible to achieve a somewhat efficient (approximately 2 log) reduction of NTM using conventional treatment lines^9^, regrowth makes treatment at this stage an effort in vain. Studies have found 10–25,000-fold increases of NTM in distribution systems compared to treatment plant outlets, even when numbers at the outlet were very low or sometimes undetectable^9,11^. Down the line, the majority of studies on domestic water systems demonstrate that plumbing systems are generally and often persistently colonized by mycobacteria, regardless of the water source, treatment, or water quality^12^. Moreover, the primary source for pulmonary infection is believed to be through the inhalation of NTM-containing aerosols, particularly from showerheads^13^. Several studies have identified identical genotypes in clinical and environmental isolates from showerhead and tap water^14^. Gebert et al.^15^ analyzed showerhead biofilm samples from 656 households across the USA and Europe and found agreement between regions where showerheads contained high abundances of two pathogenic NTM lineages (MAC and MABS) and regions with high rates of NTM lung disease. Thus, it has been proposed that NTM treatment should focus on point-of-use (POU) ultraviolet (UV) treatment rather than at the water treatment plant, in order to reduce chronic exposure to pathogenic NTM^16,17^. Some NTM species exhibit photo-repair of UV damage, which must be considered when treating drinking water or wastewater with UV radiation^18^. Furthermore, mycobacteria are known to clump ubiquitously due to their unique lipid-rich cell walls^19^. Clumps may also be expected to occur in natural waters and drinking water, due to simple aggregation or the sloughing of biofilms. Since clumping can shield microbes from UV radiation and thereby affect the perceived inactivation kinetics of that microorganism, the effect of clumping on UV treatment of NTM must be taken into account.

Recently, the UV light-emitting diode (UV-LED) has emerged as a novel UV radiation source that has the potential to replace conventional mercury UV lamps, similar to how visible LEDs are replacing fluorescent and incandescent lighting^20^. Key advantages of UV-LEDs include small size, high durability, design flexibility, ability to tailor wavelengths, no warm-up period, no mercury, and long lifetimes (achieved for UVA/UVB, and emerging for UVC LEDs)^21^. The 265 nm and 280 nm UV-LEDs are common due to the proximity of their emission peaks to that of nucleotide absorption (around 260–265 nm), while 280 nm UV-LEDs have higher electrical efficiency for practical use^22^. These characteristics make UV-LEDs suitable for traditionally infeasible applications, e.g., for POU disinfection of water in rural areas or developing countries^23^, or incorporation into a showerhead^24^ or other plumbing fixture. To design UV reactors for a certain level of disinfection performance, the fluence-response of the target microorganism to UV radiation of a particular wavelength or emission spectrum must be known—considering that different microbial species have different spectral sensitivities in the germicidal UV range^25^, it is not possible to simply use the fluence-response of a microorganism to 254 nm light from a low-pressure UV (LP UV) lamp to design an apparatus using UV-LEDs of a different wavelength or emission spectrum. Recent fluence-response data is scarce for NTM, and, to the best of our knowledge, no studies have reported the fluence-response of *M. abscessus* to any UV source. Thus, the objective of this study is to present the fluence-response of *M. abscessus* ATCC 19977 (type strain) to 280 nm UV-LED light in a water matrix. The results are expected to: (1) elucidate the UV-sensitivity of a priority pathogen, NTM, in drinking water and its treatment strategies; and (2) aid UV reactor design, especially for UV-LED disinfection units at the POU where NTM are best treated.

## Methods

### Cultivation and enumeration of *Mycobacteroides abscessus*

A pure culture of *Mycobacteroides abscessus* ATCC 19977 (American Type Culture Collection, Manassas, VA, USA) (homotypic synonym *Mycobacterium abscessus* ATCC 19977) was propagated and maintained at − 80 °C, and the second passage was used for irradiation. The stock solution was cultured in 10 mL total volume of Middlebrook 7H9 broth (Becton Dickenson, Sparks, MD, USA) with 10% (v/v) Middlebrook ADC Enrichment (Becton Dickenson, Sparks, MD, USA) and glycerol (2 mL/L). The tube was shaker-incubated at 37 °C for 46 h to late-log phase. The suspension was washed 3 times in phosphate-buffered solution (PBS, pH 7.4) and diluted to the order of 10^6^ colony-forming units per 1 mL (CFU/mL) in PBS for irradiation. The remaining concentration of viable microorganisms was determined using a CFU assay. One hundred microlitres of the irradiated solution was spread-plated in duplicate onto Middlebrook 7H10 agar plates (Becton Dickenson, Sparks, MD, USA) containing 10% (v/v) Middlebrook OADC Enrichment (Becton Dickenson, Sparks, MD, USA) and glycerol (5 mL/L). Plates were wrapped in Parafilm^®^ “M” (Bemis, Neenah, WI, USA) and incubated at 37 °C in the dark. Colonies were enumerated after 7 days.

### UV-LED apparatus

**Figure 1:**
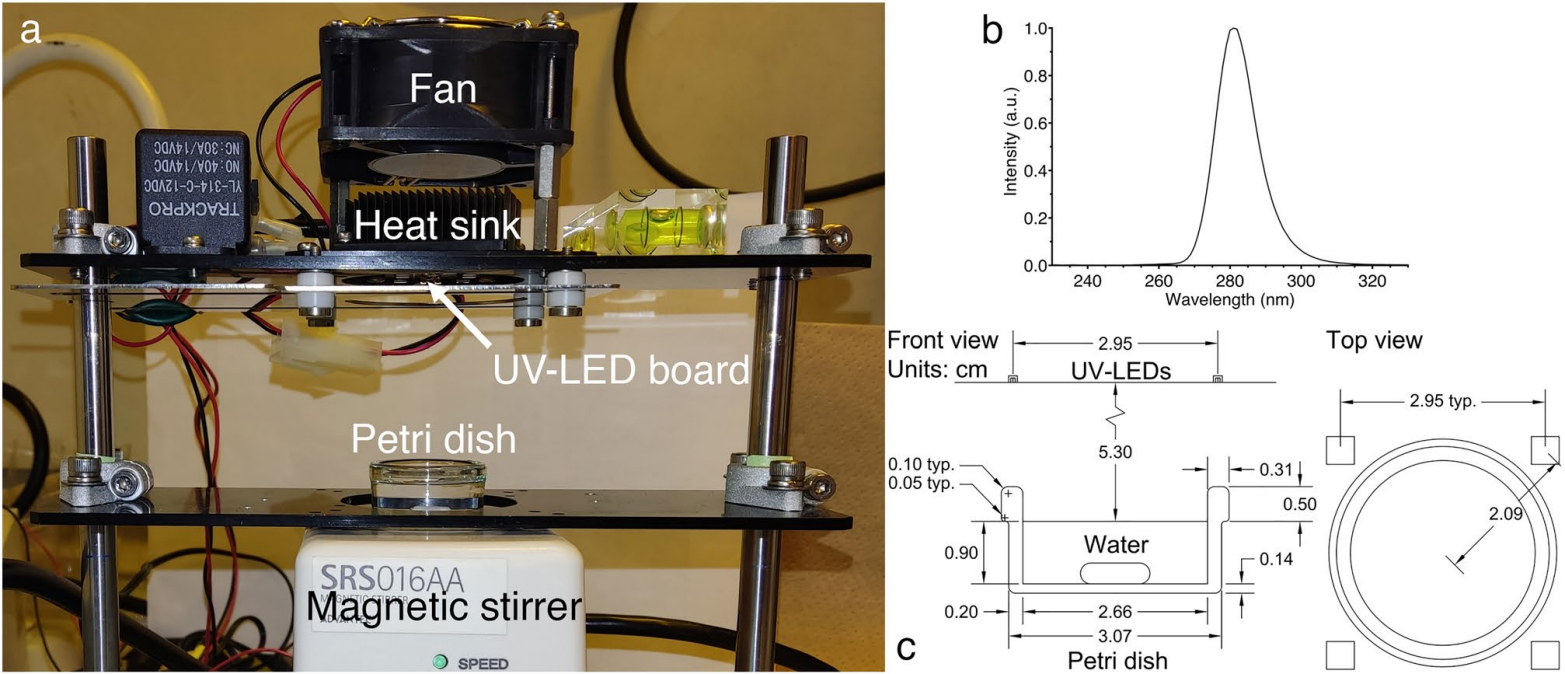
(**a**) UV-LED apparatus. (**b**) Emission spectrum of the UV-LED board, measured using a spectroradiometer. (**c**) Dimensions of the UV-LED setup under the irradiation conditions.

The UV-LED apparatus consisted of 4 downward-facing UV-LEDs mounted to a circular circuit board. The back of the circuit board contacted a heatsink with thermal paste, and a fan provided cooling. UV-LEDs (Nikkiso Giken Co. Ltd., Ishikawa, Japan) had a peak emission wavelength of 280 nm. The UV-LEDs were driven by a power supply unit providing a constant current of 544 mA at 55.1 V. Figure 1 shows the UV-LED apparatus, its dimensions under the irradiation conditions, and the UV-LED board’s emission spectrum as measured by a spectroradiometer (USR-45DA, Ushio Inc., Tokyo, Japan).

### Fluence measurement

Ferrioxalate actinometry was chosen for fluence rate determination due to the stable quantum yield of 1.25 for Fe^2+^ generation in the region of 260–340 nm^26^. The average surface fluence rate was measured to be 1.39 mW/cm^2^ following the method of Bolton and colleagues^27^. Since this value represented a surface measurement, it was adjusted by the water factor (accounting for absorbance through the solution) and divergence factor (accounting for the decrease in irradiance due to beam divergence through the solution) according to the protocol of Bolton and Linden^28^ to yield an average fluence rate of 1.06 mW/cm^2^ throughout the solution. Fluence was calculated as the product of this fluence rate and exposure time.

### UV-LED disinfection experiments

Five millilitres of the microbial PBS suspension was aliquoted into Petri dishes of 2.66 cm inner diameter, occupying a depth of 0.91 cm. Petri dishes were centered under the UV-LEDs and stirred using a small stir bar during irradiation. Dishes were irradiated for zero and specified exposure times *t* to yield N_0_ and N_t_, the CFU/mL at time zero and specified exposure time *t*, respectively. Three batches of the Petri dishes were irradiated in random order. Irradiation was performed under yellow lighting to minimize photoreactivation.

### Inactivation kinetics modelling and statistical analysis

The linear portion of the fluence-response curve was fitted to a simple log-linear model^29^ described by:

1 $$\frac{{N_{t} }}{{N_{0} }} = 10^{ - kF}$$

where *N*_*0*_ and *N*_*t*_ are as previously defined, *k* is the fluence-based inactivation rate constant (cm^2^/mJ), and *F* is the fluence (mJ/cm^2^) at time *t*.

In the case of a sigmoidal fluence-response curve, the inactivation kinetics model of Geeraerd et al.^30^, which accommodates both shoulder and tailing effects, was used for comparison:

2 $$\frac{{N_{t} }}{{N_{0} }} = 10^{ - kF} \left( {1 - \frac{{N_{res} }}{{N_{0} }}} \right)\left( {\frac{{10^{{kt_{l} }} }}{{1 + \left( {10^{{k\left( {t_{l} - t} \right)}} - 10^{ - kt} } \right)}}} \right) + \frac{{N_{res} }}{{N_{0} }}$$

where *N*_*res*_ represents a resistant subpopulation or the effect of experimental artefacts (CFU/mL), *k* is the inactivation rate constant by nonlinear fitting (cm^2^/mJ), and *t*_*l*_ is the shoulder length (mJ/cm^2^).

Regression analysis was performed using GraphPad Prism 9 (GraphPad Software, San Diego, CA, USA). For the nonlinear curve, goodness of fit was evaluated using root mean square error (RMSE), for which lower values indicate a better fit, while the coefficient of determination (R^2^) was used for the log-linear model.

## Results and discussion

### UV-sensitivity of *M. abscessus* and implications for disinfection

**Figure 2:**
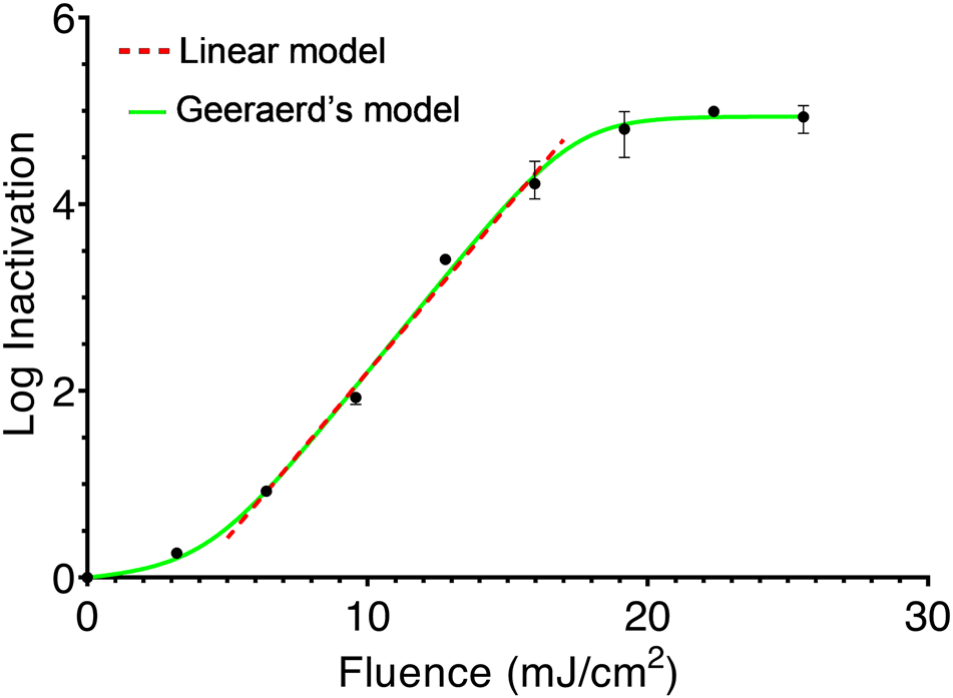
Fluence-response profile for *M. abscessus* in PBS solution exposed to 280 nm UV-LED radiation. Regression curves based on a log-linear model (Red dotted line) and Geeraerd’s model (Green line) are shown. Each data point is the mean (n = 3), and error bars represent the range of values.

The fluence-response curve of *M. abscessus* in PBS exposed to 280 nm UV-LED light is shown in Fig. 2. The results indicate that 280 nm UV-LED irradiation has a considerable germicidal effect on *M. abscessus*. Even some commercially-available ultra-compact 280 nm UV-LED POU water disinfection devices^31^, which can deliver 10–40 mJ/cm^2^, would be expected to achieve ~ 2 to > 4 log inactivation of *M. abscessus*. Studies of the distal sites of drinking water distribution systems have found total culturable mycobacteria concentrations from 10 to 3500 CFU/L^32^. We recommend that UV-LED disinfection units be designed with fluences >  ~ 15 mJ/cm^2^ to virtually eliminate the *M. abscessus* fraction.

A sigmoidal curve was observed, with “shoulder” and “tailing” areas. Our observation of shouldering is consistent with two studies^16,33^ on *M. avium* using mercury lamps. Proposed explanations for the shoulder region include: (1) the presence of clumped microorganisms, where colony formation is not disrupted until all members of the clump become inactivated; (2) a condition where the rate of resynthesis of a vital component exceeds the rate of its destruction^34^; and (3) the necessity to destroy a large number of one or more types of critical components (single-hit multiple-target phenomenon)^35^. Reasons for tailing may include the existence of a resistant subpopulation or experimental artefacts (*N*_*res*_), including again the clumping effect^30^. Coohill and Sagripanti^36^ found that tailing was mitigated for up to 6 log inactivation in the same bacteria once clumping was controlled for. Bohrerova and Linden^37^ reported enhanced UV disinfection efficiency of wastewater suspensions of *M. terrae* that had large particles removed by filtration. Mycobacteria are particularly prone to clumping in culture due to their thick, waxy cell walls^38–40^, and significant clumping has been observed in suspensions of *M. abscessus* from both solid and liquid media^41,42^. Thus, clumping was likely a cause of the shouldering and tailing phenomena exhibited in this study. The shoulder length (*t*_*l*_) was 4.1 mJ/cm^2^, which was more than those previously reported^22^ at 280 nm for *Escherichia coli* and *Pseudomonas aeruginosa* (1.95 mJ/cm^2^ and 1.01 mJ/cm^2^, respectively) but less than that of *Bacillus subtilis* spores (8.24 mJ/cm^2^). Interestingly, the inactivation rate constant of *M. abscessus* (discussed below) also falls in between those of the aforementioned microorganisms, suggesting a possible correlation between shoulder length and UV-sensitivity.

### Inactivation kinetics modelling

The model parameters, measures of goodness of fit, and fluences corresponding to 1, 2, 3, and 4 log inactivation are shown in Table 1. The log-linear model was fitted only to the linear part of the curve and yielded an R^2^ value of 0.984. Accommodating the overall sigmoidal shape, a better fit was provided by Geeraerd’s model, with an RMSE of 0.138.

**Table 1:** Results of regression analysis using log-linear and Geeraerd’s models. Brackets contain 95% confidence intervals.

Parameter	Log-linear model	Geeraerd’s model
Inactivation rate constant, *k* (cm^2^/mJ)	0.356 (0.323–0.388)	0.372 (0.345–0.404)
N_res_ (CFU/mL)	Not applicable	101.4 (78.50–130.1)
t_l_ (mJ/cm^2^)	Not applicable	4.095 (3.493–4.676)
R^2^	0.984	Not applicable
RMSE	Not applicable	0.138
Fluence for 1 log inactivation (mJ/cm^2^)	6.624	6.609
Fluence for 2 log inactivation (mJ/cm^2^)	9.435	9.448
Fluence for 3 log inactivation (mJ/cm^2^)	12.25	12.16
Fluence for 4 log inactivation (mJ/cm^2^)	15.06	14.98

The *k* value reflects the UV-sensitivity of microorganisms. Since fluence is the product of fluence rate (generally constant in a batch experiment) and exposure time, both fluence-based (cm^2^/mJ) and time-based (s^−1^) inactivation rate constants can be defined. In this discussion and comparison, only fluence-based inactivation rate constants will be used. Since the fluence-response of *M. abscessus* to UV has not been previously reported to the best of our knowledge, studies of MAC, other NTM, and reference microorganisms in drinking water disinfection were chosen for comparison. The comparison of *k* values from recent studies is shown in Table 2. For completeness, data for similar UV-LED wavelengths as well as LP UV (monochromatic at 253.7 nm) and medium-pressure UV (MP UV; polychromatic in the wavelength range of around 185–600 nm) were included when no 280 nm UV-LED data were available for the microorganism. Since UV-sensitivity depends considerably on wavelength due to different absorption by nucleotides and other biomolecules^43^, it is inappropriate to directly compare *k* values for dissimilar wavelengths. Based on studies employing a similar methodology^22,44^, the *k*_280_ (fluence-based inactivation rate constant at 280 nm) values indicate that *M. abscessus* is more resistant than *P. aeruginosa* and *L. pneumophila* and much less resistant than adenovirus, MS2 coliphage, bacteriophage Qβ, and *B. subtilis* spores. The *k*_280_ of *M. abscessus* in this study was approximately 0.64 times that of *E. coli* (*k*_280_ = 0.56 cm^2^/mJ, *k*_254_ = 0.81 cm^2^/mJ)^22^. According to Hayes et al.^16^, the average *k*_254_ from three *M. avium* isolates was 0.41 cm^2^/mJ, which is about 0.51 times the *k*_254_ reported for *E. coli* by Rattanakul and Oguma^22^. Shin et al.^45^ reported for two *M. avium* isolates a *k*_254_ of 0.17 cm^2^/mJ, which is 0.21 times that reported by Rattanakul and Oguma^22^ for *E. coli*, noting that their isolates were much more resistant than most protozoan parasites and pathogenic bacteria, and comparable to many pathogenic viruses but not as resistant as rotavirus or adenovirus. Accounting for methodological differences and assuming a certain similarity in UV-sensitivity within NTM, our results appear consistent with those from *M. avium* studies and the overall sensitivity pattern of reference drinking water pathogens.

**Table 2:** Comparison of fluence-based inactivation rate constants from recent UV inactivation studies for selected NTM and microorganisms relevant to drinking water disinfection.

Microorganism	Wavelength (nm)	Fluence-based inactivation rate constant, *k* ± 95% CI (cm^2^/mJ)	Reference
Adenovirus type 5 ATCC VR5	285	0.023 ± 0.003	^44^
MS2 coliphage ATCC 15597-B1	285	0.029 ± 0.000	^44^
*Bacteriophage Qβ*	280	0.056 ± 0.001	^22^
*Bacillus subtilis spores* ATCC 6633	280	0.104 ± 0.006	^22^
*M. terrae* ATCC 15755	LP UV	0.16	^18^
*M. terrae* ATCC 15755	MP UV	0.17	^18^
*M. avium* subsp. *Hominissuis*, HMC02 (white transparent) (WT)	LP UV	0.17	^45^
*M. avium* subsp. *Hominissuis*, HMC02 (white opaque) (WO)	LP UV	0.17	^45^
*M. avium* subsp. *Hominissuis*, HMC02 (WT)	MP UV	0.18	^45^
*M. avium* subsp. *Hominissuis*, HMC02 (WO)	MP UV	0.20	^45^
*M. abscessus* ATCC 19977	280	0.356 ± 0.033 (log-linear model)	This study
*M. intracellulare* ATCC13950	LP UV	0.27	^16^
*M. intracellulare* B12CC2	LP UV	0.36	^16^
*M. avium* D55A01	LP UV	0.34	^16^
*M. avium* 33B	LP UV	0.43	^16^
*M. avium* W41	LP UV	0.46	^16^
*L. pneumophila*	280	0.453 ± 0.013	^22^
*P. aeruginosa*	280	0.511 ± 0.053	^22^
*Escherichia coli* IFO 3301	280	0.561 ± 0.039	^22^

*M*. Mycobacterium.

Considering the microorganisms typically used for UV reactor validation, it was difficult to identify a surrogate microorganism with both similar UV sensitivity and fluence-response curve (i.e., exhibiting shoulder and tailing) to *M. abscessus*. Although its inactivation kinetics is log-linear without shouldering or tailing, T1UV, a double-stranded DNA virus commonly used as a surrogate for reactor validation, exhibits comparable UV sensitivity to *M. abscessus*. Ward et al.^46^ found a log-linear fluence-response curve for T1UV with a *k*_254_ of 0.23 cm^2^/mJ, which suggests that T1UV is somewhat less sensitive to UV than *M. abscessus* (*k*_280_ = *0.*356 cm^2^/mJ). Beck et al.^25^ reported fluences (± 95% CI) of 4.3 (± 0.4), 8.5 (± 0.9), 12.8 (± 1.3), and 17.0 (± 1.8) mJ/cm^2^ to achieve 1, 2, 3, and 4 log inactivation, respectively, of T1UV using a 253.7 nm tunable laser. The group also provided the relative spectral sensitivity (action spectrum) of T1UV, showing that the UV sensitivity of T1UV to 253.7 nm and 280 nm radiation is similar. The fluences for 1 and 2 log inactivation of T1UV are slightly lower than those for *M. abscessus*, and those for 3 and 4 log inactivation are slightly higher than those for *M. abscessus*. This change may be due to shouldering in the *M. abscessus* fluence-response curve, which may result in lower perceived UV sensitivity at low fluences. Further studies, particularly with controls for clumping, should be performed to confirm the suitability of T1UV as a surrogate microorganism for *M. abscessus* at 280 nm.

### Control of mycobacteria by physical versus chemical disinfectants and strategies at the POU

The susceptibility of mycobacteria and selected emerging pathogens to LP UV, free chlorine, chloramines, ozone, and chlorine dioxide was compared by Jacangelo et al.^47^. The data showed that mycobacteria (represented by *M. avium* and *M. fortuitum*) are among the most resistant microbes across nearly all disinfectants, but especially so for the chemical disinfectants. Taylor et al.^48^ studied the effects of chlorine, chloramine, chlorine dioxide, and ozone on five *M. avium* isolates and found, for chlorine, CT_99.9%_ (the product of the disinfectant concentration (in parts per million) and the time (in minutes) to 99.9% inactivation) values ranging from 51 to 204. These values were 580–2300 times higher than those for *E. coli*. For chlorine dioxide and ozone, CT_99.9%_ values were over 100- and 50-fold higher (respectively) than those for *E. coli*. Clumping was controlled for, and they concluded that most of the *M. avium* strains were highly resistant to the four chemical disinfectants studied. Such high CT values suggest that chemical disinfection is not an effective method for controlling mycobacteria. In contrast, our results and the literature indicate that mycobacteria can be effectively inactivated at common UV disinfection fluences (typically 40 mJ/cm^2^ in large-scale reactors, and 10–40 mJ/cm^2^ in some UV-LED POU water disinfection devices).

In the context of OPPPs and POU drinking water treatment, several strategies centered around the POU have been proposed to reduce exposure to NTM and NTM-laden aerosols^49^. These include: draining water heaters, increasing water heater temperature to 55 °C, switching from piped to well water, installing microbiological filters (≤ 0.2 μm pore size), avoiding the use of granular activated carbon (GAC) filters (which promote the growth of NTM without preventing their passage), replacing showerheads with ones having large holes, disinfecting showerheads monthly, reducing bathroom aerosols, removing aerators from taps, and boiling water for 10 min. Many of these strategies are inefficient, time-consuming, possibly infeasible, or may introduce new issues, and most only reduce the risk of NTM exposure to some uncertain degree. Recent studies on POU disinfection methods for NTM generally consider filtration- or UV-based methods as the most feasible, efficient, and low-cost^50^. Falkinham^49^ notes that water filters with ≤ 0.2 μm pore sizes will prevent the passage of NTM, and tap and showerhead replacement filters have been available mainly for the hospital market. However, the filters should be replaced every 30 days and are costly (e.g., $50–100/month). Similar commercially-available 0.2 μm in-line filters were shown by Norton et al.^50^ to prevent the passage of *M. smegmatis* for the rated filter lifetimes, while a GAC system failed to significantly reduce *M. abscessus* and *M. avium* numbers. Using a mixed suspension of tap water-adapted *M. abscessus, M. avium*, and *M. chimaera*, the group also evaluated a hollow-fiber, two-stage membrane filtration system, which prevented passage of the mycobacterial suspension for the entire 68-day evaluation period. Finally, two different water bottle UV sterilization systems, “Mountop” and “SteriPEN,” were challenged with the same mixed suspension and UV treated according to manufacturers’ guidelines once daily for 7 days, followed by once weekly treatment for up to 56 days. Both UV systems achieved a > 4 log reduction in the mixed suspension CFU after 4 days of daily treatment, although the difference between treated and control bottles was only significant for the SteriPEN system. This was likely due to immersion of the SteriPEN device, whereas the Mountop lamp was located in the bottle lid. Mixed suspension and biofilm-associated CFU were assayed after 56 days, and a > 4 log reduction was maintained in both bottles. Both UV systems used a small LP UV mercury lamp with a 90 s cycle, and a single treatment significantly reduced the bacterial burden in either bottle (0.8–1.8 log). Since fluence rate was not determined, it is difficult to compare their results to the current study. Overall, the effectiveness of both in-line POU and batch systems for controlling NTM in a convenient, accessible, and practical manner has been clearly demonstrated. The results of this study are expected to improve the design of UV-LED disinfection units.

## Conclusions

One advantage of this study over previous NTM UV disinfection research is the high number of fluences tested, which produced a well-defined fluence-response curve. Shoulder and tailing phenomena, which are difficult to analyze in studies involving fewer fluences^45^, were clearly expressed. These details improved model fitting and allowed a better understanding and characterization of the UV inactivation kinetics of NTM. A limitation of this study is the lack of investigation into photoreactivation, whereas POU-treated water may be shortly exposed to room light or sunlight that triggers photoreactivation. For *M. avium*, McCarthy and Schaefer^33^ reported both photoreactivation, after 30 min–3 h of visible light exposure following UV irradiation, and dark repair, while Hayes et al.^16^ found no photoreactivation following simultaneous UV and fluorescent light irradiation and no dark repair. For *M. terrae*, Bohrerova and Linden^18^ evaluated photo-repair under 30 min exposure to full-spectrum lighting following LP UV or MP UV irradiation. Photo-repair of 0.4–10% and 16–43% by CFU viability assay and endonuclease sensitive site (ESS) assay, respectively, were reported, with no statistically significant difference between LP UV and MP UV irradiation. Due to differences in methodologies, further research is needed to ascertain whether and which NTM species perform photoreactivation. In our experiment, while irradiation was performed under dimmed yellow lighting to minimize photoreactivation, dilution and plating were performed in fluorescent room lighting, and the exposure time for each batch was similar. If photoreaction were to have occurred, the results would still be conservative. Another limitation is the lack of control for clumping. Barr et al.^51^ recently reported that clumping of mycobacteria was a major determinant of CFU count and could not be disrupted by vortexing, sonication, or centrifugation, as measured by flow cytometry (FCM). Thus, while vortexing was performed for at least several seconds before each manipulation during experiments, more potent methods might better disrupt clumping and affect the shoulder and tailing phenomena.

This study used a batch system with Petri dishes containing mixed, non-flowing solutions of microorganisms of known concentrations with known fluences, whereas real-world UV reactors and POU disinfection units are generally flow-through systems. The results can be applied to such flow-through systems by first determining the system’s reduction equivalent fluence (REF) (mJ/cm^2^), the UV fluence delivered by a UV reactor as determined by a biodosimetry test, under a set of operating conditions. Then, the fluence-response curve from this study can be used to estimate the expected inactivation of *M. abscessus* using that reactor under those operating conditions. Even at the initial design stage, if fluence is estimated using computer modeling, the curve can again provide the expected inactivation. Thus, the results can be used to design or estimate the performance of real-world UV disinfection units.

In conclusion, 280 nm UV-LEDs are effective for the disinfection of *M. abscessus* in water, with 2 log inactivation expected at a fluence of 10 mJ/cm^2^, which is technically possible to deliver even with commercially-available compact apparatuses for POU applications. Inactivation kinetics modelling showed that *M. abscessus* is more resistant than *P. aeruginosa* and *L. pneumophila*, suggesting that NTM are among the most UV-resistant OPPPs, and further research might lead to appropriate surrogate microorganisms for reactor validation. Our results can be used to inform UV-LED disinfection unit design against these important pathogens in drinking water systems.

## Data Availability

This article contains no supplementary information. The data that support the findings of this study are included in this article; further inquiries can be directed to the corresponding author.
